# A 24-Year-Old Woman With Acute Lower Abdominal Pain

**DOI:** 10.1016/j.acepjo.2026.100402

**Published:** 2026-04-22

**Authors:** Adrianna Salah, Jami Zajicek, Naofal da Silva, Alexandra Jackson, Joseph Pepe

**Affiliations:** 1 Department of Emergency Medicine, Lincoln Memorial University–DeBusk College of Osteopathic Medicine, Harrogate, Tennessee, USA; 2 Department of General Surgery, AdventHealth Tampa, Tampa, Florida, USA; 3 Department of Emergency Medicine, University of South Florida–Morsani College of Medicine, Tampa, Florida, USA; 4 Department of Emergency Medicine, AdventHealth Tampa, Tampa, Florida, USA

**Keywords:** endometrioma, endometriosis, hemorrhagic ascites, ovarian torsion mimic, acute abdominal pain

## 1 Case Presentation

A 24-year-old woman with a history of chronic right-sided sciatic pain presented to the emergency department with acute-onset periumbilical and lower abdominal pain, accompanied by nausea and vomiting. On examination, the abdomen was soft but tender in the mid to lower regions, moderately distended, with voluntary guarding. A large, soft, and compressible mass was palpated, extending to the periumbilical region. Laboratory evaluation was notable for leukocytosis.

**Figure 1:**
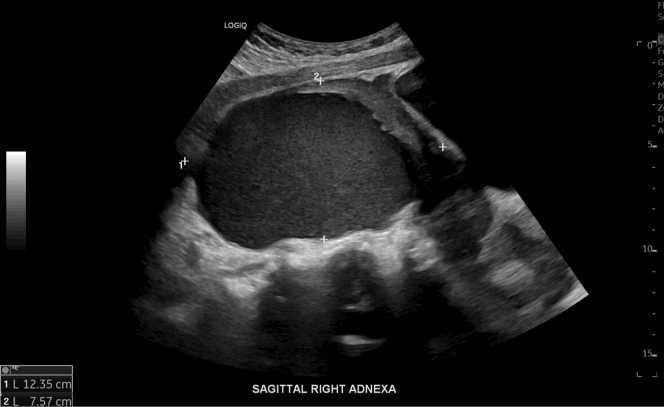
Transabdominal pelvic ultrasound demonstrating a large, complex hypoechoic mass in the right adnexa.

Pelvic ultrasound exhibited a large, complex hypoechoic cystic mass within the right adnexal region measuring approximately 12 cm, with peripheral vascular flow and trace pelvic free fluid (Fig 1).

**Figure 2:**
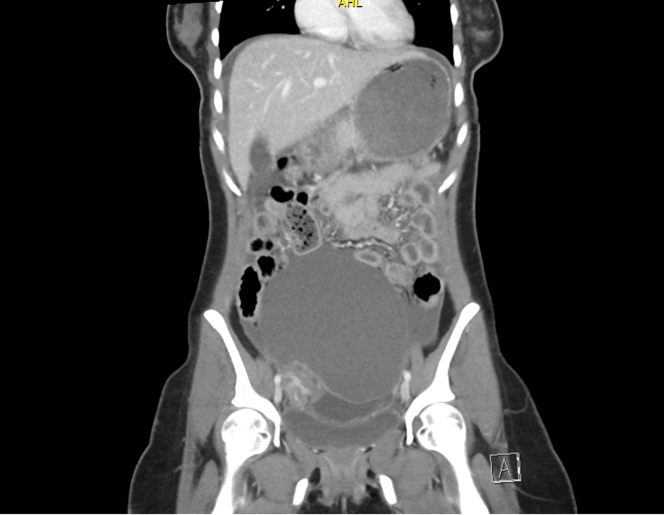
Coronal computed tomography of the abdomen and pelvis with intravenous contrast demonstrating a large lesion abutting the right adnexa measuring 12.6 cm.

**Figure 3:**
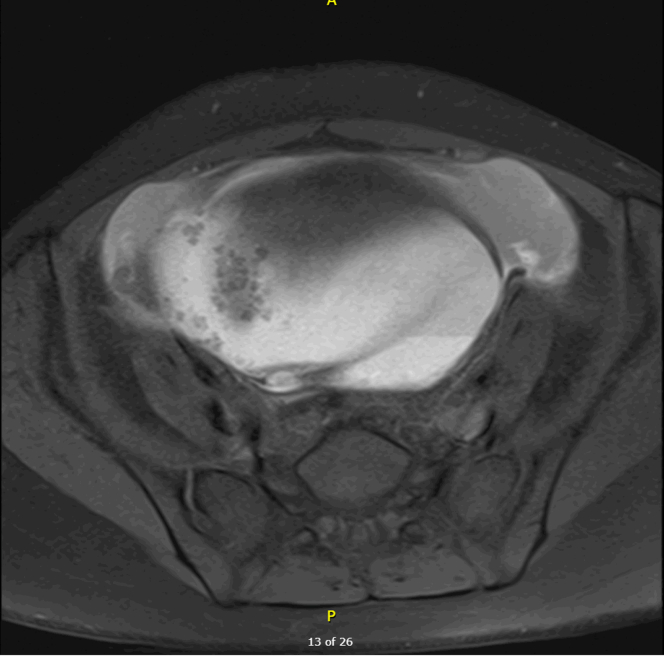
Magnetic resonance imaging of the pelvis, T1 fat suppressed, demonstrating mural nodules and endometriomas.

Contrast-enhanced computed tomography of the abdomen and pelvis demonstrated a large cystic lesion measuring 12.6 cm with associated small-volume ascites (Fig 2). Subsequent magnetic resonance imaging showed multiple right adnexal endometriomas, pelvic endometriotic implants, and hemorrhagic ascites, findings most consistent with advanced endometriosis (Fig 3). Ovarian torsion and hemorrhagic neoplasm were considered but deemed less likely. Additionally, elevated CA-125 and CA 19-9 were noted. Gynecology was consulted, and the patient was admitted for further evaluation and subsequent surgical management.

## 2 Diagnosis

### 2.1 Large Right Ovarian Endometriomas With Hemorrhagic Ascites Mimicking Ovarian Torsion

Large ovarian endometriomas may present as complex adnexal masses with ascites and elevated serum markers, closely mimicking ovarian torsion or malignancy in the emergency setting. Serum markers may be elevated in benign endometriosis, but lack diagnostic specificity and should not be interpreted in isolation.^1^ Advanced disease can produce hemorrhagic cysts, adhesions, and extra-ovarian implants with atypical or referred pain patterns.^2^^,^^3^ This case highlights the importance of recognizing imaging features of endometriosis and correlating findings across imaging modalities to guide appropriate consultation and management.

## Funding and Support

By *JACEP Open* policy, all authors are required to disclose any and all commercial, financial, and other relationships in any way related to the subject of this article as per ICMJE conflict of interest guidelines (see www.icmje.org). The authors have stated that no such relationships exist. Publication costs were supported by Lincoln Memorial University - Debusk College of Osteopathic Medicine.

## Conflict of Interest

All authors have affirmed they have no conflicts of interest to declare.
